# Detection of collagen band–associated regions in H&E-stained colonic biopsies of collagenous colitis patients using superpixel-based feature extraction and neural network classification

**DOI:** 10.1186/s13000-026-01783-x

**Published:** 2026-03-28

**Authors:** Vytautas Kiudelis, Robertas Petrolis, Rima Ramonaitė, Dainius Jančiauskas, Lina Poškienė, Juozas Kupčinskas, Povilas Šabanas, Reda Čerapaitė-Trušinskienė, Diana Meilutytė-Lukauskienė, Algimantas Kriščiukaitis

**Affiliations:** 1 Department of Gastroenterology, Lithuanian University of Health Sciences, Kaunas, LT 50161 Lithuania; 2 Institute for Digestive Research, Lithuanian University of Health Sciences, Kaunas, LT 44307 Lithuania; 3 Neuroscience Institute, Lithuanian University of Health Sciences, Kaunas, LT 50009 Lithuania; 4 Department of Physics, Mathematics and Biophysics, Lithuanian University of Health Sciences, Kaunas, LT 50009 Lithuania; 5 Department of Pathology, Lithuanian University of Health Sciences, Kaunas, LT 50009 Lithuania; 6 Lithuanian Energy Institute, Kaunas, LT 44403 Lithuania

**Keywords:** Collagenous colitis (CC), Collagen, Histopathology, Superpixels, Deep learning, Machine-learning-based assistance system

## Abstract

**Background:**

Collagenous colitis (CC) is diagnosed histologically and is characterised by a thickened subepithelial collagen band together with inflammatory and epithelial changes. Although routine haematoxylin and eosin (H&E) staining is sufficient for diagnosis in most cases, visual assessment of the collagen band can be challenging in borderline or heterogeneous specimens. Additional stains may be required in diagnostically difficult situations.

**The aim:**

To develop a machine-learning–based algorithm for detecting subepithelial collagen band-associated regions in routine H&E-stained colonic biopsy images as a decision-support tool for histopathological assessment.

**Methods:**

H&E-stained colonic biopsy specimens from 36 patients with histologically confirmed CC were imaged at 20 × magnification (1392 × 1040 pixels). Images were segmented into 1,000 superpixels using the Simple Linear Iterative Clustering (SLIC) algorithm. Superpixels overlapping with expert-provided rough annotations of the collagen band were labelled and characterised using normalised RGB histograms. A feed-forward neural network classifier (three hidden layers, 10 neurons per layer) was trained to distinguish collagen band–associated from non-collagen regions. Class imbalance was addressed by data augmentation of minority-class superpixels. Post-processing with connected-component size filtering was applied to enforce spatial continuity. Superpixel-level performance was evaluated quantitatively, and image-level outputs were assessed using expert acceptability scoring.

**Results:**

The classifier achieved a superpixel-wise accuracy of 0.928 (sensitivity 0.898, specificity 0.953). Size-based post-processing substantially reduced isolated false-positive detections. At the image level, the final algorithm achieved an acceptability accuracy of 0.846 according to expert evaluation. The model successfully highlighted subepithelial collagen band–associated regions consistent with expert annotations but did not model additional diagnostic features required for complete CC diagnosis.

**Conclusion:**

Our superpixel-based neural network highlights collagen-rich regions in H&E-stained colonic biopsies, offering decision support for pathologists. As diagnosis of collagenous colitis requires broader histopathological and clinical context, this method is intended as a decision-support tool rather than a stand-alone diagnostic solution.

## Introduction

Collagenous colitis (CC) is a chronic inflammatory disorder of the large intestine, clinically characterised by non-bloody watery diarrhoea, often accompanied by faecal incontinence, nocturnal symptoms, abdominal discomfort, and unintended weight loss, all of which significantly impair patients’ quality of life [1–3]. Although traditionally considered a benign condition, long-term prospective data show that nearly half of patients experience a relapsing or chronically active disease course, with persistent symptoms and reduced health-related quality of life even during clinical remission [1, 3]. The pathogenesis of CC is multifactorial, involving a dysregulated mucosal immune response, epithelial barrier dysfunction, alterations in the gut microbiome, and potential genetic predisposition, although the underlying aetiology remains incompletely understood [4].

Epidemiological studies from Europe and North America consistently show a marked rise in the incidence of microscopic colitis – including CC, lymphocytic colitis (LC), and their incomplete forms, with some regions now reporting incidence and prevalence rates comparable to those of classic inflammatory bowel diseases [5–7]. CC usually affects older adults, with a marked female predominance, but it can also occur in younger adults and, in rare instances, even in children [4]. Although incidence appears to have stabilised in some populations in recent years, overall prevalence continues to increase, reflecting both improved disease recognition and a genuine rise in disease burden [5–7]. These clinical and epidemiological insights highlight the need for accurate, reproducible, and accessible diagnostic tools to support timely evaluation and effective management of CC. Diagnosing CC can be challenging because endoscopic findings are typically normal or near normal, which limits the ability of endoscopy alone to raise diagnostic suspicion [8]. Even with high-definition endoscopes, only a minority of patients show macroscopic abnormalities, such as mucosal lacerations, erythema, attenuation of the vascular pattern, or surface textural changes, and these findings are often subtle and inconsistently reported across studies [8]. As a result, clinical suspicion remains crucial for prompting biopsy acquisition. In recognition of this, European guidelines recommend systematic sampling from both the right and left colon to minimise the risk of missed diagnoses and to ensure adequate histological assessment [2]. Histopathological examination remains the cornerstone of diagnosis. CC is defined by the presence of a thickened subepithelial collagen band measuring ≥ 10 µm, accompanied by increased inflammatory infiltrates within the lamina propria, whereas a band thickness of 5–10 µm is considered indicative of incomplete CC [2, 9]. Routine haematoxylin and eosin (H&E) staining is sufficient for most cases, while special stains or immunohistochemistry may assist in ambiguous situations by enhancing visualisation of the subepithelial collagen band [9].

Although interobserver agreement among gastrointestinal pathologists is generally high when distinguishing microscopic colitis (MC) from non-MC, significant variability arises when subclassifying MC and identifying incomplete forms of the disease. Previous studies have demonstrated excellent agreement (exceeding 88–91%) when pathologists distinguish MC or MCi from non-MC conditions such as inflammatory bowel disease or normal mucosa [10, 11]. However, concordance declines substantially when differentiating among MC subtypes, particularly in borderline or incomplete cases. κ values of only 0.60–0.75 have been reported for interobserver agreement across five diagnostic categories (CC, LC, MCi, inflammatory bowel disease, and normal), highlighting the difficulty of reliably separating incomplete microscopic colitis (MCi) from fully developed CC or LC using routine H&E-stained sections [10]. These findings were corroborated by Limsui et al., who observed similarly reduced agreement when pathologists attempted finer-grained histological classification, despite excellent reproducibility in distinguishing MC from non-MC overall [11]. Taken together, these data indicate that the diagnostic process becomes less reproducible when evaluating subtle collagen band thickening, borderline histological changes, or heterogeneous tissue morphology. This variability underscores the need for objective, reproducible, and efficient decision-support tools to assist pathologists, particularly in cases where histopathological features are subtle, overlapping, or affected by technical artefacts.

Automated histopathological image analysis has advanced significantly over the past decade. Traditional computational approaches have enabled quantitative analysis of inflammatory bowel disease, neuroblastoma, melanoma, and various cancers [12–17], but these methods often rely on handcrafted features, are sensitive to staining variability, and require extensive task-specific parameter tuning, which limits their generalisability across datasets. More recently, deep learning has transformed digital pathology by learning hierarchical, data-driven representations directly from raw images, improving performance in tissue classification, biomarker identification, disease subtype discrimination, and outcome prediction [18–20]. Complementary machine learning-assisted biophysical methods, such as mid-infrared spectrochemical imaging and photoacoustic spectral analysis, have further expanded the ability to quantify collagen in fibrotic or neoplastic tissues [21–23].

The diagnosis of CC requires histological confirmation before starting immunosuppressive therapy, such as budesonide, which is considered first-line treatment based on multiple randomised controlled trials [24–29]. As up to half of CC patients experience a relapsing or chronically active disease course [1, 30], consistent and timely histopathological evaluation is essential for guiding management.

In this study, we developed a machine learning-based algorithm that integrates the Simple Linear Iterative Clustering (SLIC) superpixel segmentation method with neural network classification to detect subepithelial collagen band-associated regions in routine H&E-stained colonic biopsy images. The method operates directly on standard H&E slides without requiring collagen-specific staining and is trained using rough, region-level expert annotations, thereby reducing annotation burden while preserving morphologically meaningful structures. Importantly, the proposed approach is not intended to provide an automated diagnosis of collagenous colitis. Histopathological diagnosis requires integration of multiple criteria, including collagen band thickness, surface epithelial injury, goblet cell alterations, and intraepithelial lymphocytosis. Subepithelial collagen thickening alone is not entirely specific and may occur in other reactive or inflammatory contexts. Therefore, the objective of this work is to develop a computational decision-support tool that highlights collagen band–associated regions as a visual aid within the broader diagnostic workflow, particularly in visually demanding or heterogeneous cases.

## Materials and methods

### Study cohort

Thirty-six patients with active CC were included in this study. The protocol was approved by the Kaunas Regional Biomedical Research Ethics Committee (Protocol No. P1-BE-2–31/2018), and all participants provided written informed consent. Eligible patients had undergone a colonoscopy for chronic watery diarrhoea and were diagnosed with CC according to establish international histological criteria [2].

**Fig. 1:**
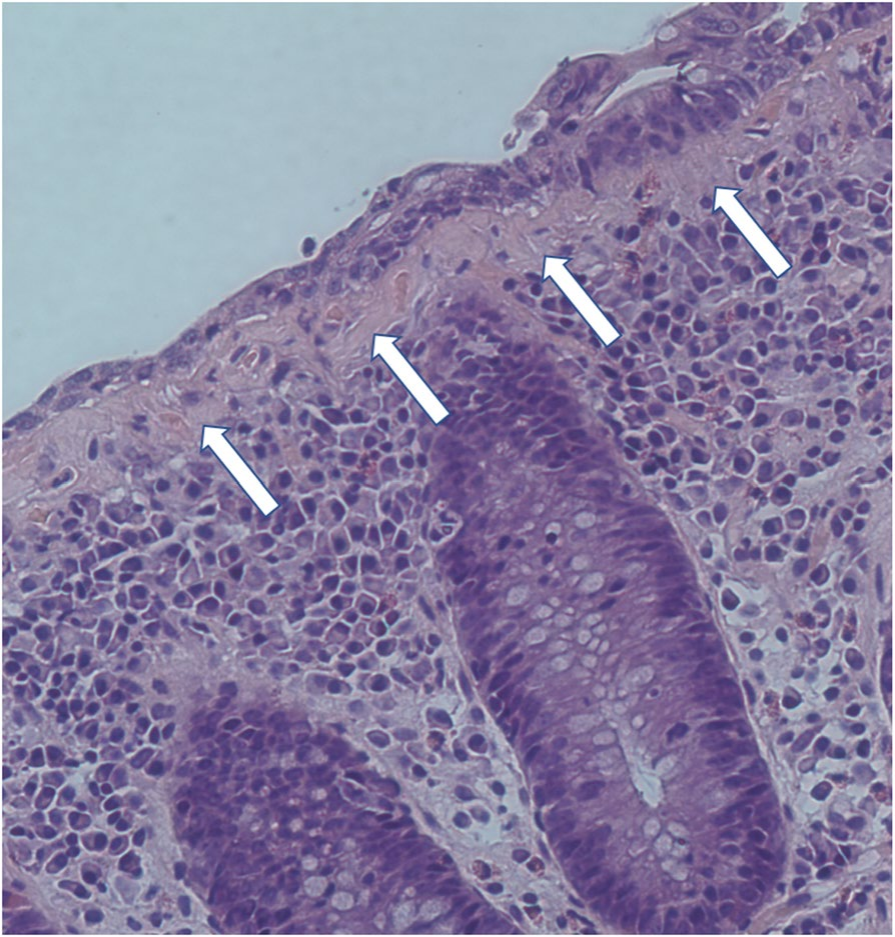
Histological section of collagenous colitis (H&E, 20x)

Active disease was defined according to the Hjortswang criteria as at least three stools per day or at least one watery stool per day [31]. Representative H&E-stained colonic biopsy specimens were selected for digital imaging (Fig. 1). In total, 1,700 histological images were acquired and used subsequently computational analysis.

### Histological specimen imaging

Colour images were obtained using an OLYMPUS IX71 light microscope equipped with a Q IMAGING EXI Aqua camera at 20 × magnification. Images were captured at a resolution of 1392 × 1040 pixels (0.6 µm per pixel). Figure 1 shows a representative 20 × micrograph of CC used in this analysis.

### Image preprocessing

All preprocessing, segmentation and machine learning procedures were implemented in MATLAB R2023b (MathWorks, Natick, MA, USA) and executed on a computational workstation with an AMD Ryzen Threadripper 3970X 32-core processor (3.70 GHz) and 128 GB RAM.

As image acquisition was performed over an extended period, variations in illumination intensity and colour balance led to inter-image variability. To reduce these effects, colour normalisation was applied independently to each image in RGB colour space before segmentation and feature extraction. Illumination inhomogeneity was corrected using morphological opening with a structuring element larger than typical cellular structures, thereby estimating and removing low-frequency background intensity variations. After background correction, histogram alignment was applied channel-wise to standardise colour intensity distributions across images, following the method described by Petrolis et al. [32].

All preprocessing parameters were fixed before model training and applied uniformly across the dataset to avoid per-image tuning and minimise bias. Preprocessing was performed independently for each image and did not use information from other images, thus preventing data leakage between training and validation subsets. The purpose of this step was to reduce technical variability while preserving underlying morphological structures relevant to collagen band detection.

### Superpixel segmentation and feature extraction

The proposed algorithm was designed to analyse human colonic histological specimen images containing both normal tissue architecture and areas of increased subepithelial collagen deposition. As preparing large training datasets with pixel-precise annotations is time-consuming and labour-intensive, rough, region-level annotations provided by expert pathologists were used to label the training data.

**Fig. 2:**
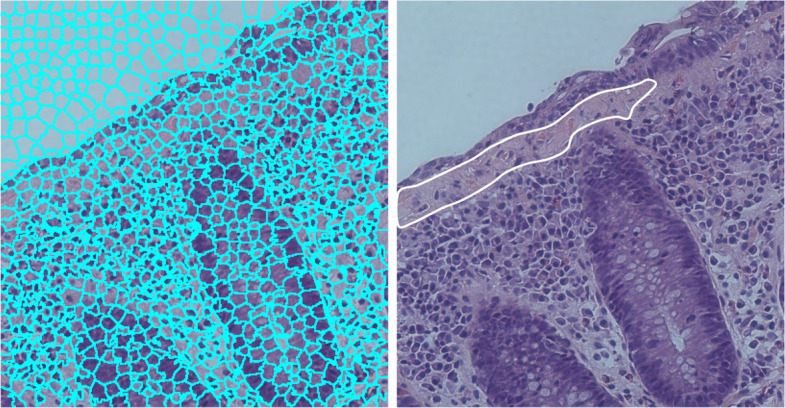
Superpixel segmentation (left) and expert-provided rough annotation of the thickened subepithelial collagen layer (right)

H&E-stained colonic tissue typically comprises visually homogeneous regions, including epithelium, lamina propria, inflammatory infiltrates, and collagen-rich zones. To exploit this property, superpixel segmentation was applied to group adjacent pixels with similar colour and texture characteristics, thereby preserving local structural boundaries and improving segmentation stability in complex histological images (Fig. 2). Superpixel segmentation was performed using the MATLAB superpixels function, which implements the SLIC algorithm [33].

To execute the SLIC algorithm, the desired number of superpixels (k) must be specified. The algorithm computes an approximate grid interval (S) that determines the linear dimension of each superpixel, as follow:

1 $$S=\sqrt{\frac Nk}$$

where S is the grid interval for each superpixel, N is the total number of pixels in the image, and k is the number of equally sized superpixels to be generated. This relationship ensures approximately uniform superpixel size and spatial distribution across the image.

Following preliminary image analysis, dividing each analysed image (1392 × 1040 pixels) into 1,000 superpixels was found to provide an optimal balance between boundary adherence and computational efficiency. This superpixel size is large enough to reduce sensitivity to imaging artefact with and small enough to capture the complex morphological features characteristic of CC pathology. By grouping superpixels with similar properties, the algorithm could effectively segment the thickened subepithelial collagen layer, which was the primary target of the analysis.

**Fig. 3:**
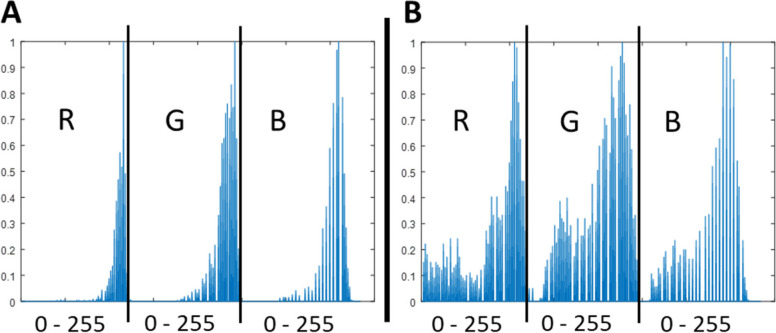
Typical normalized superpixel value histogram in RGB colour space (**A** – CC group, **B** – normal tissue)

The core concept of the algorithm was to identify CC-related morphological features by optimising superpixel groupings and analysing the colour distribution of regions representing key histopathological characteristics. To classify superpixels as normal or pathological (indicating areas of increased subepithelial collagen deposition (Fig. 3)), normalised RGB pixel value histograms were used as classification features.

Superpixels within CC-affected regions typically showed higher RGB intensity values and lower value dispersion compared with superpixels representing normal tissue. These differences reflect tissue structural changes associated with a thickened subepithelial collagen layer. Despite image preprocessing, superpixel histogram patterns varied across specimens, and direct image thresholding, discriminant analysis, or cluster analysis did not achieve satisfactory classification performance.

Therefore, a fully connected feed-forward neural network classifier was selected. The network was implemented in the MATLAB environment using fitcnet function [34]. Network architecture was selected through automated hyperparameter optimization, which identified a three-hidden-layer architecture as optimal. Although, the optimal neuron counts per layer varied within a narrow range (4–13 neurons), a uniform configuration of ten neurons per hidden layer was adopted as a parsimonious and stable architecture providing comparable predictive performance. Hidden layers used hyperbolic tangent (tanh) activation functions, and a softmax function was applied in the output layer to estimate class probabilities. Model parameters were optimized by minimising the cross-entropy loss function using the limited-memory Broyden–Fletcher–Goldfarb–Shanno (L-BFGS) algorithm. Input features were standardized prior to training.

We anticipated that histogram patterns and local variations characteristic of CC-affected areas would be reliably captured by a trained neural network.

### Dataset preparation and augmentation

Neural network performance is strongly influenced by dataset size, as large datasets reduce overfitting and improve robustness to noise and outliers. From the histologically analysed images, 65,664 superpixels covering CC-affected tissue and 230,137 superpixels representing normal tissue were obtained. Superpixels were assigned to the CC-affected class when at least 70% of their area overlapped with expert-annotated collagen regions; this criterion ensured reliable class labelling rather than reflecting a biological threshold. Superpixels corresponding to background or white areas without tissue were excluded using adaptive thresholding.

Because of class imbalance, with fewer CC-labelled superpixels, data augmentation was applied to the minority class. Random noise (mean = 0; range = – 0.15 to 0.15) was added to the RGB histogram values of CC-affected superpixels, followed by renormalisation. The noise range was constrained to preserve the overall histogram shape and class-discriminative characteristics while improving robustness to small input perturbations.

After augmentation, the final dataset consisted of 436,508 normalised superpixel histograms, including 206,371 CC-affected superpixels and 230,137 normal tissue superpixels. During network training, the dataset was randomly partitioned into training (80%) and validation (20%) subsets. Image-level performance evaluation using expert acceptability scoring was performed separately, as described in the Result section.

## Results

### Superpixel-level classification performance

The feed-forward neural network was trained on the full augmented dataset of 436,508 superpixel histograms. Training took 29 min and achieved a superpixel-wise classification accuracy of 0.928. The model demonstrated high sensitivity (0.898) and specificity (0.953), indicating that most collagen-associated (“disease”) superpixels were correctly detected while maintaining a low false-positive rate in normal tissue superpixels.

**Fig. 4:**
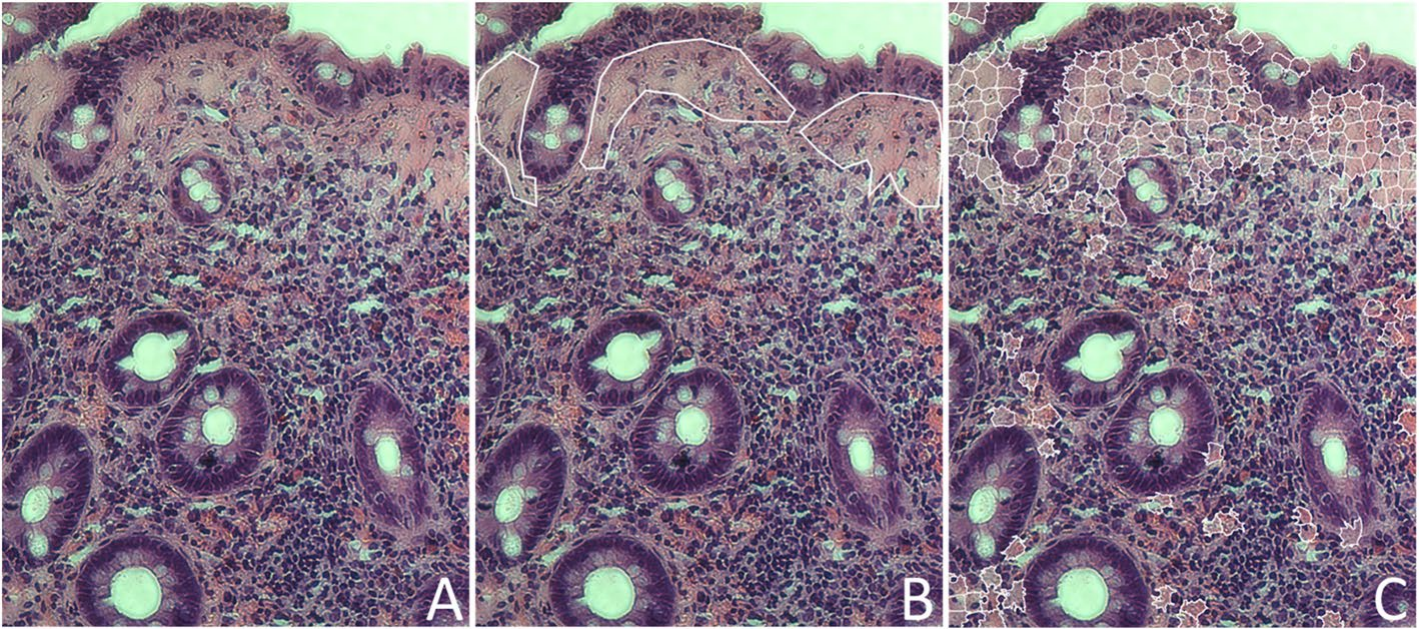
Representative model output at the superpixels level: **A** original histological image; **B** expert rough annotation of the thickened subepithelial collagen layer; (**C**) superpixels classified as “disease” by the neural network

To qualitatively assess model behaviour, representative images were visually inspected by comparing expert-marked collagen regions with model outputs. Figure 4 shows a typical example: the original H&E image (Fig. 4A), the expert’s rough annotation of thickened subepithelial collagen (Fig. 4B), and the corresponding superpixels classified as “disease” by the model (Fig. 4C). The model output showed good spatial correspondence with the annotated subepithelial collagen band but also produced scattered isolated “disease” superpixels outside the expected subepithelial location.

### Post-processing using connected-component size filtering

From a histopathological perspective, CC is characterised by a continuous or near-continuous thickened subepithelial collagen band, rather than small, isolated collagen-positive “islands” dispersed throughout the lamina propria. During expert review, isolated single “disease” superpixels or small disconnected clusters were therefore interpreted as likely segmentation artefacts rather than biologically plausible collagen band detections.

**Fig. 5:**
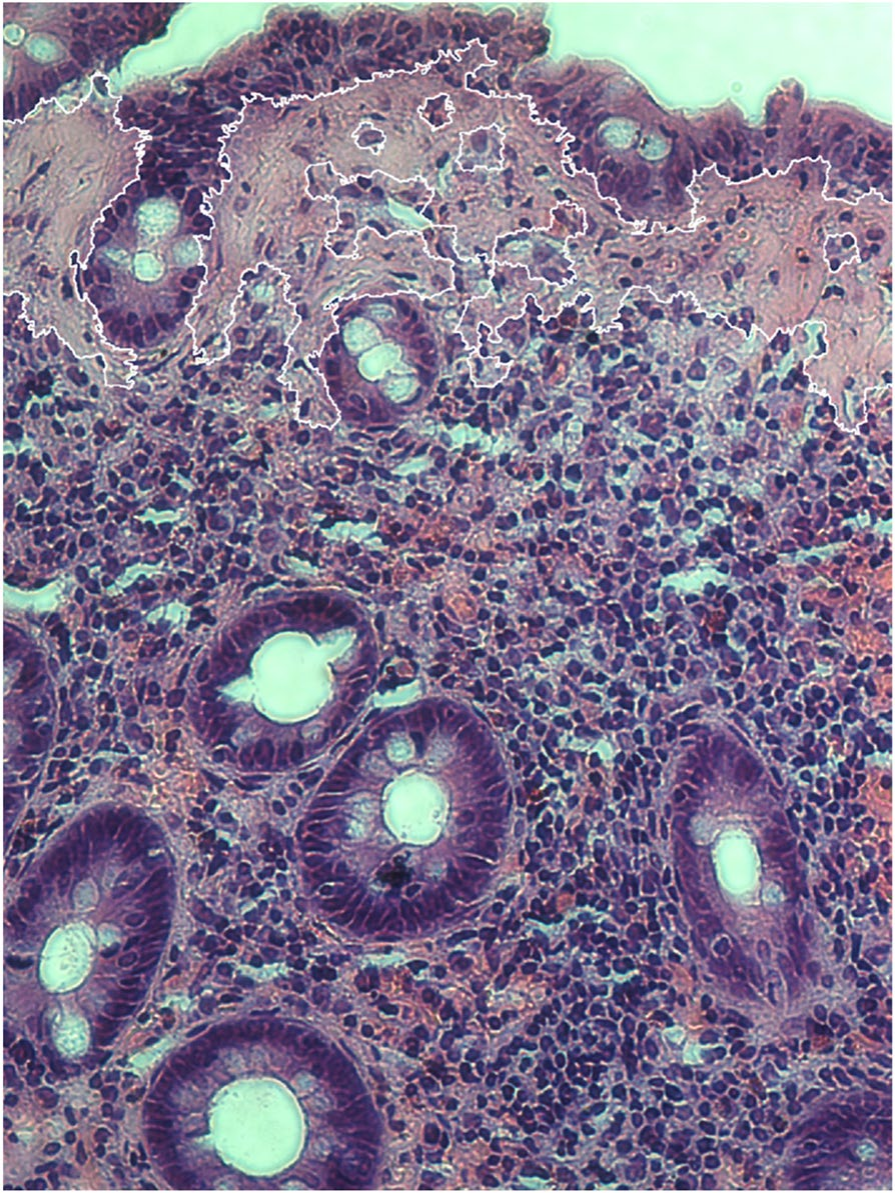
Detected collagen-rich region after post-processing (size-filtered connected “disease” superpixel components)

To reduce these false-positive fragments, a connected-component size filter was incorporated into the workflow. Specifically, regions containing fewer than ten connected “disease” superpixels (approximately 10,000 pixels) were removed. This step substantially improved the plausibility of the segmented collagen region by enforcing minimal spatial continuity consistent with CC morphology. Figure 5 shows the detected collagen area after applying this size filter, demonstrating a more coherent and anatomically consistent subepithelial band.

### Image-level validation by expert acceptability scoring

After applying the size filter, model performance was evaluated at the image level to reflect practical diagnostic utility. For each test image, the classifier was trained on the remaining images and then applied to the withheld image (leave-one-out scheme). The resulting collagen segmentation masks were presented to histology experts for qualitative assessment. Each output was labelled as acceptable (1) or unacceptable (0) based on whether the CC-affected collagen region was appropriately highlighted.

**Fig. 6:**
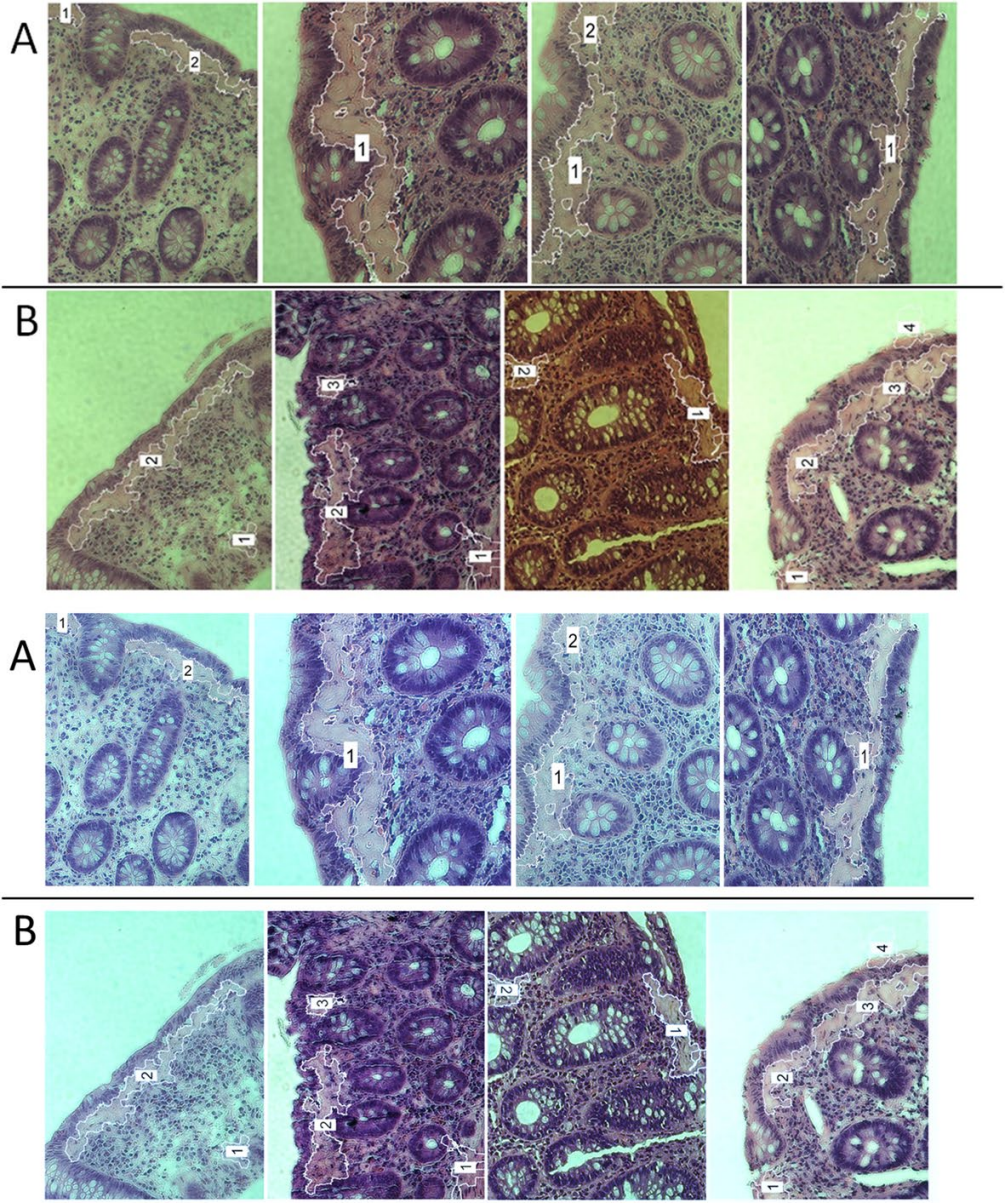
Representative segmentation examples of raw images: **A** correctly segmented images; **B** incorrectly segmented images. Outlines indicate the final detected collagen region after filtering

Using this expert acceptability endpoint, the final algorithm achieved an image-level accuracy of 0.846. Figure 6 shows examples of typical outputs. In correctly segmented cases (Fig. 6A), the algorithm delineated collagen-rich subepithelial regions in a manner consistent with expert expectations. In incorrectly segmented cases (Fig. 6B), the segmentation included regions not compatible with subepithelial collagen deposition, resulting in false-positive detection patterns.

### Failure modes and qualitative error analysis

Visual inspection of incorrect cases indicated that algorithm failures were most often linked to suboptimal slide or image quality, including tissue disruption, folding or tearing artefacts, uneven staining, or poor focus. In these situations, structures such as blood vessels, muscle tissue, or empty or artefactual spaces could display colour distributions similar to those of collagen-rich regions and were sometimes misclassified as “disease.” These findings suggest that robustness to preparation artefacts and staining variability remains a key determinant of performance and may be improved in future work through stricter quality-control filtering, inclusion of artefact examples during training, or integration of spatial or architectural constraints beyond histogram-only features.

## Discussion

Computational analysis of histopathological images has evolved from classical quantitative methods to modern deep learning–based frameworks that support diagnostic assessment across a wide range of diseases. Earlier approaches enabled systematic evaluation and abnormality detection in ulcerative colitis, neuroblastoma, melanoma, and various malignancies [12–17]. However, traditional pipelines often rely on handcrafted features and task-specific parameter tuning, making them sensitive to staining variability and imaging differences, which limits their generalisability across tissue types and acquisition settings. In contrast, deep learning methods can learn hierarchical, data-driven representations directly from image-derived inputs and have shown strong performance in tissue classification, biomarker discovery, outcome prediction, and disease subtype discrimination [18–20]. Additionally, machine-learning-assisted biophysical techniques, such as mid-infrared spectrochemical imaging and photoacoustic spectral analysis, have shown promise for quantitative collagen assessment in fibrotic and neoplastic tissues [21–23]. Nevertheless, many collagen-focused computational methods depend on non-routine modalities or specialised staining, which may reduce clinical accessibility.

Histological confirmation remains essential for diagnosing CC, placing the pathologist at the centre of the diagnostic workflow. While interobserver agreement is excellent when distinguishing microscopic colitis (MC) from non-MC (91–98%) [10, 11], reproducibility decreases substantially when separating CC and LC from incomplete forms, with agreement reported at only 59–67% [10]. These diagnostically challenging cases often involve subtle, discontinuous, or heterogeneous collagen band thickening, contributing to diagnostic uncertainty. In this context, computer-assisted tools may help improve reproducibility and reduce the time required for visually demanding assessments. In this study, we developed a machine-learning–assisted approach for detecting collagen-rich regions in routine H&E-stained light microscopy images. The proposed pipeline was intentionally designed for simplicity and rapid training-set creation, using rough, region-level expert annotations rather than pixel-precise labelling. This design reduces annotation burden while still enabling learning at the level of meaningful morphological units (superpixels). At the superpixel level, the neural network demonstrated strong discrimination between collagen-associated and normal regions, and the post-processing size filter improved biological plausibility by removing isolated detections inconsistent with the typical morphology of the subepithelial collagen band. Importantly, final performance evaluation incorporated expert judgement of image-level segmentation acceptability, providing a clinically oriented endpoint beyond purely numerical classification metrics.

Our results are consistent with previous work aiming to computationally support CC diagnosis, while differing in key practical aspects. Malekian et al. used a Multi-Layer Perceptron model with colour and texture features to segment the subepithelial collagen band, reporting excellent performance, although their analysis was restricted to selected regions [35]. Fiehn et al. developed an automated image analysis approach applied to full histological slides, including incomplete CC cases and controls, and reported good agreement with pathologists [36]. In comparison, our method achieved comparable qualitative segmentation performance while operating directly on routine H&E-stained images, avoiding additional specimen preparation with collagen-specific stains such as Masson’s Trichrome or Van Gieson [35, 36].

Although collagen-specific stains accentuate the subepithelial collagen band and facilitate automated measurement, their use introduces additional complexity into the diagnostic workflow, including extra slide preparation, increased laboratory costs, extended turnaround time, and additional expert effort. In routine clinical practice, H&E staining is sufficient for diagnosing CC in most cases, and special stains are typically reserved for diagnostically challenging or borderline situations. In contrast to VG-based automated image analysis approaches that aim to quantify collagen band thickness, the present study deliberately focused on routine H&E-stained sections. While this design inevitably resulted in slightly lower segmentation accuracy, it substantially reduced the need for additional staining, specialised infrastructure, and extensive expert annotation. From a clinical perspective, this trade-off may be acceptable when the primary goal is to support screening, highlight suspicious regions, or assist pathologists in cases with subtle or heterogeneous histological features, rather than to replace detailed morphometric assessment.

This study has several limitations. First, the algorithm focuses exclusively on detecting subepithelial collagen band–associated regions and does not model additional histopathological features required for a complete diagnosis of collagenous colitis, such as surface epithelial injury, goblet cell depletion, or intraepithelial lymphocytosis. Subepithelial collagen thickening alone is not specific and may also be observed in other reactive or inflammatory contexts. Therefore, the proposed method should be interpreted strictly as a decision-support tool rather than as a stand-alone diagnostic classifier. Second, the study cohort comprised only patients with histologically confirmed collagenous colitis, and no control group was included. Consequently, diagnostic specificity across different disease categories, including normal mucosa, lymphocytic colitis, incomplete microscopic colitis, or other inflammatory conditions, could not be assessed. Validation in larger, multicentre cohorts including appropriate control groups will be essential to determine generalisability and clinical applicability. Third, segmentation performance was influenced by specimen and imaging quality. Tissue artefacts, uneven staining, section folds, tearing, and suboptimal focus contributed to false-positive detections in structures such as blood vessels, muscle tissue, or empty artefactual spaces. In addition, the use of RGB histogram features may be sensitive to residual colour variation between laboratories and imaging systems. Standardised acquisition protocols, automated quality-control procedures, and multicentre validation will therefore be important steps in future development. Finally, the current method does not incorporate crypt orientation assessment or quantitative collagen band thickness measurement, both of which may be relevant for distinguishing incomplete collagenous colitis and for comparison with guideline-based diagnostic thresholds [2, 9]. Integration of quantitative morphometric features may further improve specificity and diagnostic interpretability.

Future work should extend this framework to multi-feature modelling that integrates additional histological parameters, including epithelial injury, goblet cell density, and inflammatory cell distribution. Incorporating spatial constraints related to luminal proximity and automated estimation of crypt orientation and collagen band thickness may further improve diagnostic utility. Validation in multicentre datasets, including normal biopsies and other forms of microscopic colitis, will be essential to determine generalisability and clinical applicability.

Overall, this study demonstrates that a pragmatic combination of SLIC superpixels, histogram-based feature extraction, neural network classification, and simple post-processing can highlight collagen-band-associated regions consistent with CC on routine H&E-stained microscopy images. The proposed algorithm is intended as a decision-support tool to assist pathologists by highlighting areas warranting closer inspection or consideration of additional staining. It is not designed to replace comprehensive histopathological assessment, which incorporates epithelial injury, inflammatory features, and clinical correlation. By operating on standard H&E slides without requiring special stains, this approach may provide aid in the pathological diagnosis of CC, particularly in resource-limited settings.

## Conclusions

Our superpixel-based neural network identifies collagen-associated regions in H&E-stained colonic biopsies and may assist pathologists in evaluating CC. Trained using coarse, region-level expert annotations, the model reduces annotation burden while maintaining meaningful segmentation performance and supporting more standardized assessment. As CC diagnosis requires integration of clinical and histopathological findings that involve more than just subepithelial collagen band thickness, the system is intended as a decision-support tool rather than a stand-alone diagnostic solution.

## Data Availability

The datasets used and analysed during the current study are available from the corresponding author on reasonable request.

## Abbreviations

CC: Collagenous colitis
H&E: Haematoxylin and eosin
LC: Lymphocytic colitis
MC: Microscopic colitis
SLIC: Simple Linear Iterative Clustering

## References

[1] Verhaegh BPM, Münch A, Guagnozzi D, Wildt S, Cebula W, Pedersen N, et al. The long-term disease course of microscopic colitis: a European prospective incident cohort study. J Crohns Colitis. 2025;19(7):jjaf110. 10.1093/ecco-jcc/jjaf110 40583189

[2] Miehlke S, Guagnozzi D, Zabana Y, Tontini GE, Fiehn AMK, Wildt S, et al. European guidelines on microscopic colitis: United European Gastroenterology (UEG) and European Microscopic Colitis Group (EMCG) statements and recommendations. United European Gastroenterol J. 2020; 10.1177/2050640620951905 PMC8259259 33619914

[3] Nyhlin N, Wickbom A, Montgomery SM, Tysk C, Bohr J. Long-term prognosis of clinical symptoms and health-related quality of life in microscopic colitis: a case-control study. Aliment Pharmacol Ther. 2014;39(9):963–72. 24612051 10.1111/apt.12685

[4] Burke KE, D’Amato M, Ng SC, Pardi DS, Ludvigsson JF, Khalili H. Microscopic colitis. Nat Rev Dis Primers. 2021. 10.1038/s41572-021-00273-2. 34112810 10.1038/s41572-021-00273-2

[5] Tome J, Sehgal K, Kamboj AK, Harmsen WS, Kammer PP, Loftus EV, et al. The epidemiology of microscopic colitis in Olmsted county, Minnesota: population-based study from 2011 to 2019. Clin Gastroenterol Hepatol. 2022;20(5):1085–94. 34216819 10.1016/j.cgh.2021.06.027 PMC8716639

[6] Weimers P, Ankersen DV, Lophaven S, Bonderup OK, Münch A, Løkkegaard ECL, et al. Incidence and prevalence of microscopic colitis between 2001 and 2016: a Danish nationwide cohort study. J Crohns Colitis. 2021;14(12):1717–23. 10.1093/ecco-jcc/jjaa108 32502240

[7] Tong J, Zheng Q, Zhang C, Lo R, Shen J, Ran Z. Incidence, prevalence, and temporal trends of microscopic colitis: a systematic review and meta-analysis. Am J Gastroenterol. 2015;110(2):265–76. 10.1038/ajg.2014.431. 25623658 10.1038/ajg.2014.431

[8] Marlicz W, Skonieczna-Żydecka K, Yung DE, Loniewski I, Koulaouzidis A. Endoscopic findings and colonic perforation in microscopic colitis: A systematic review. Vol. 49, Digestive and Liver Disease. Elsevier B.V.; 2017. p. 1073–85. 10.1016/j.dld.2017.07.015 28847471

[9] Langner C, Aust D, Ensari A, Villanacci V, Becheanu G, Miehlke S, et al. Histology of microscopic colitis-review with a practical approach for pathologists. Vol. 66, Histopathology. Blackwell Publishing Ltd; 2015. p. 613–26. 10.1111/his.12592 25381724

[10] Fiehn AMK, Bjørnbak C, Warnecke M, Engel PJH, Munck LK. Observer variability in the histopathologic diagnosis of microscopic colitis and subgroups. Hum Pathol. 2013;44(11):2461–6. 24029708 10.1016/j.humpath.2013.06.004

[11] Limsui D, Pardi DS, Smyrk TC, Abraham SC, Lewis JT, Sanderson SO, et al. Observer variability in the histologic diagnosis of microscopic colitis. Inflamm Bowel Dis. 2009;15(1):35–8. 18623168 10.1002/ibd.20538

[12] Petrolis R, Ramonaite R, Jančiauskas D, Kupčinskas J, Pečiulis R, Kupčinskas L, et al. Digital imaging of colon tissue: method for evaluation of inflammation severity by spatial frequency features of the histological images. Diagn Pathol. 2015;10(1):159. 10.1186/s13000-015-0389-7 PMC4570696 26370784

[13] Orlov NV, Weeraratna AT, Hewitt SM, Coletta CE, Delaney JD, Mark Eckley D, et al. Automatic detection of melanoma progression by histological analysis of secondary sites. Cytometry Part A. 2012;81 A(5):364–73. 10.1002/cyto.a.22044 PMC3331954 22467531

[14] Sertel O, Catalyurek UV, Shimada H, Guican MN. Computer-aided prognosis of neuroblastoma: detection of mitosis and karyorrhexis cells in digitized histological images. In: 2009 Annual International Conference of the IEEE Engineering in Medicine and Biology Society. IEEE; 2009. p. 1433–6. 10.1109/IEMBS.2009.5332910 19963746

[15] Trivizakis E, Ioannidis GS, Souglakos I, Karantanas AH, Tzardi M, Marias K. A neural pathomics framework for classifying colorectal cancer histopathology images based on wavelet multi-scale texture analysis. Sci Rep. 2021;11(1):15546. 10.1038/s41598-021-94781-6 PMC8324876 34330946

[16] Yu E, Monaco JP, Tomaszewski J, Shih N, Feldman M, Madabhushi A. Detection of prostate cancer on histopathology using color fractals and Probabilistic Pairwise Markov models. In: 2011 Annual International Conference of the IEEE Engineering in Medicine and Biology Society. IEEE; 2011. p. 3427–30. 10.1109/IEMBS.2011.6090927 22255076

[17] Sieren JC, Weydert J, Bell A, De Young B, Smith AR, Thiesse J, et al. An automated segmentation approach for highlighting the histological complexity of human lung Cancer. Ann Biomed Eng. 2010;38(12):3581–91. 20571856 10.1007/s10439-010-0103-6 PMC2996273

[18] Komura D, Ishikawa S. Machine learning methods for histopathological image analysis. Vol. 16, Computational and Structural Biotechnology Journal. Elsevier B.V.; 2018. p. 34–42. 10.1016/j.csbj.2018.01.001 PMC6158771 30275936

[19] Acs B, Rantalainen M, Hartman J. Artificial intelligence as the next step towards precision pathology. Vol. 288, Journal of Internal Medicine. Blackwell Publishing Ltd; 2020. p. 62–81. 10.1111/joim.13030 32128929

[20] Cooper M, Ji Z, Krishnan RG. Machine learning in computational histopathology: Challenges and opportunities. Vol. 62, Genes Chromosomes and Cancer. John Wiley and Sons Inc; 2023. p. 540–56. 10.1002/gcc.23177 37314068

[21] Maknuna L, Kim H, Lee Y, Choi Y, Kim H, Yi M, et al. Automated structural analysis and quantitative characterization of scar tissue using machine learning. Diagnostics. 2022;12(2):534. 10.3390/diagnostics12020534 PMC8871086 35204623

[22] Adi W, Rubio Perez BE, Liu Y, Runkle S, Eliceiri KW, Yesilkoy F. Machine learning-assisted mid-infrared spectrochemical fibrillar collagen imaging in clinical tissues. J Biomed Opt. 2024;29(09). Available from: https://www.spiedigitallibrary.org/journals/journal-of-biomedical-optics/volume-29/issue-09/093511/Machine-learning-assisted-mid-infrared-spectrochemical-fibrillar-collagen-imaging-in/10.1117/1.JBO.29.9.093511.full. 10.1117/1.JBO.29.9.093511 PMC11448345 39364328

[23] Li J, Bai L, Chen Y, Cao J, Zhu J, Zhi W, et al. Detecting collagen by machine learning improved photoacoustic spectral analysis for breast cancer diagnostics: feasibility studies with murine models. J Biophotonics. 2024;18:e202400371 10.1002/jbio.202400371 PMC11700697 39600191

[24] Baert F, Schmit A, D’Haens G, Dedeurwaerdere F, Louis E, Cabooter M, et al. Budesonide in collagenous colitis: a double-blind placebo-controlled trial with histologic follow-up. Gastroenterology. 2002;122(1):20–5. 11781276 10.1053/gast.2002.30295

[25] Bonderup OK, Hansen JB, Birket-Smith L, Vestergaard V, Teglbjærg PS, Fallingborg J. Budesonide treatment of collagenous colitis: a randomised, double blind, placebo controlled trial with morphometric analysis. Gut. 2003;52(2):248–51. 12524408 10.1136/gut.52.2.248 PMC1774966

[26] Miehlke S, Heymer P, Bethke B, Bästlein E, Meier E, Bartram HP, et al. Budesonide treatment for collagenous colitis: A randomized, double-blind, placebo-controlled, multicenter trial. Gastroenterology. 2002;123(4):978–84. 12360457 10.1053/gast.2002.36042

[27] Miehlke S, Madisch A, Kupcinskas L, Petrauskas D, Böhm G, Marks HJ, et al. Budesonide is more effective than mesalamine or placebo in short-term treatment of collagenous colitis. Gastroenterology. 2014;146(5):1222-1230.e2. 10.1053/j.gastro.2014.01.019. 24440672 10.1053/j.gastro.2014.01.019

[28] Münch A, Bohr J, Miehlke S, Benoni C, Olesen M, Öst Å, et al. Low-dose budesonide for maintenance of clinical remission in collagenous colitis: a randomised, placebo-controlled, 12-month trial. Gut. 2016;65(1):47–56. 25425655 10.1136/gutjnl-2014-308363 PMC4717436

[29] Bonderup OK, Hansen JB, Teglbjárg PS, Christensen LA, Fallingborg JF. Long-term budesonide treatment of collagenous colitis: a randomised, double-blind, placebocontrolled trial. Gut. 2009;58(1):68–72. 18669576 10.1136/gut.2008.156513

[30] Verhaegh BPM, Münch A, Guagnozzi D, Wildt S, Cebula W, Diac AR, et al. Course of disease in patients with microscopic colitis: a European prospective incident cohort study. J Crohns Colitis. 2021;15(7):1174–83. 33433605 10.1093/ecco-jcc/jjab007

[31] Hjortswang H, Tysk C, Bohr J, Benoni C, Kilander A, Larsson L, et al. Defining clinical criteria for clinical remission and disease activity in collagenous colitis. Inflamm Bowel Dis. 2009;15(12):1875–81. 19504614 10.1002/ibd.20977

[32] Petrolis R, Cizas P, Borutaite V, Krisciukaitis A. Method of fluorescence imaging for evaluation of membrane potential in cultured neurons using transmembrane voltage sensitive dye. In: Biomedical engineering 2011: Proceedings of International Conference. 2011. p. 16–9.

[33] Achanta R, Shaji A, Smith K, Lucchi A, Fua P, Süsstrunk S. SLIC superpixels compared to state-of-the-art superpixel methods. IEEE Trans Pattern Anal Mach Intell. 2012;34(11):2274–82. 22641706 10.1109/TPAMI.2012.120

[34] MathWorks. MATLAB Help Center. 2024. fitcnet. Train neural network classification model. Available from: https://www.mathworks.com/help/stats/fitcnet.html. Cited 2025 Apr 28.

[35] Malekian V, Amirfattahi R, Sadri S, Mokhtari M, Aghaie A, Rezaeian M. Computer aided measurement of sub-epithelial collagen band in colon biopsies for collagenous colitis diagnosis. Micron. 2013;45:59–67. 23200274 10.1016/j.micron.2012.10.015

[36] Fiehn AMK, Kristensson M, Engel U, Munck LK, Holck S, Engel PJH. Automated image analysis in the study of collagenous colitis. Clin Exp Gastroenterol. 2016;8(9):89–95. 10.2147/CEG.S101219 PMC4833367 27114713

